# Healthy Social Network Use and Well-Being during Adolescence: A Biopsychosocial Approach

**DOI:** 10.3390/children10101649

**Published:** 2023-10-03

**Authors:** Tania Gaspar, Marina Carvalho, Catarina Noronha, Fábio Botelho Guedes, Ana Cerqueira, Margarida Gaspar de Matos

**Affiliations:** 1 HEI-LAB, Digital Human-Environment Interaction Labs, Lusófona University, Campo Grande 376, 1749-024 Lisbon, Portugal; 2 Institute of Environmental Health (ISAMB), Faculty of Medicine, University of Lisbon, Av. Professor Egas Moniz, 1649-028 Lisbon, Portugal; marina.carvalho@ismat.pt (M.C.); catarinanoronha@edu.ulisboa.pt (C.N.); anacerqueira@edu.ulisboa.pt (A.C.); mmmatos@ucp.pt (M.G.d.M.); 3 University Hospital Center of Algarve, Manuel Teixeira Gomes Higher Institute, Dr. Estêvão de Vasconcelos no 33 A, 8500-656 Portimão, Portugal; 4 Faculty of Human Kinetics, University of Lisbon/Lisbon University, Estrada da Costa, 1499-002 Oeiras, Portugal

**Keywords:** social networks, dependence, well-being, adolescents, ecological approach

## Abstract

Screen time and the use of social networks is the most frequent form of leisure time occupation and socializing for adolescents. The present study is aimed at understanding and characterizing, from an ecological perspective, what distinguishes healthy and less healthy or even dependent use of social media in young people and the influence on adolescents’ well-being. This paper is based on the Health Behaviour in School-aged Children (HBSC) from 2022, a survey carried out every 4 years, in collaboration with the World Health Organisation (WHO), following an international protocol. A total of 7643 students from the 6th, 8th, 10th, and 12th school grades responded, of which 53.9% were female, with an average age of 15.05 (*SD* = 2.36). The sample is representative of the school grades under study. The results allow us to study and identify similarities and differences between three groups related to the level of healthiness in the use of social networks and its relations to well-being from an ecological perspective. What distinguishes adolescents with less healthy use of social networks is that they are more often female, older, have more self-injurious behaviour, higher alcohol consumption, and a worse relationship with teachers. The adolescents with the highest level of dependence on social networks are those who have a higher perception of lack of safety at school and in their area of residence, as well as a higher use of screen time as a leisure activity. The well-being of adolescents using social media in a healthy way is explained by fewer psychological symptoms, better stress management strategies, better body awareness, more physical activity, less time online with friends, and better relationships with family and teachers. Technologies and social networks are important for the well-being of adolescents; it is essential to promote a healthy, critical and balanced use with other “screen-free” activities and to promote socio-emotional skills, a lack of which seems to be one of the biggest risk factors associated with the healthy use of technologies.

## 1. Introduction

Social media use (SMU) has become an integrated part of daily life, leading to several studies on its impact on mental health and well-being, particularly in adolescents. Also, at this developmental stage, family and friend networks, in the real and the virtual world, represent a major role in normative development, as they satisfy adolescents’ needs for social support [1,2,3]. However, despite the existence of several studies on the negative impact of SMU on mental health [4], its positive outcomes are still understudied [5].

Well-being, defined as a state of positive mental health and wellness [6], is of upmost relevance throughout one’s lifespan. From an ecological perspective, there are different individual and contextual factors which can positively or negatively affect adolescents’ well-being which, in turn, is associated with a range of life outcomes. Contextual factors, such as parent and peer support [7,8,9,10], and individual factors, such as gender, psychological symptoms, and physical activity [11,12,13] have been found to predict well-being in adolescents.

The effects of SMU on well-being have also been studied [14,15] and these effects seem to depend on the type of use. In a systematic review carried out to understand the association between SMU according to four domains (time spent, activity, investment, and addiction), Keles and colleagues [15] found that the included studies evidenced a positive correlation between all the domains of SMU with depression, anxiety, and psychological distress. However, in an umbrella review carried out by Valkenburg [16] on the impact of SMU on well-being and ill-being in adolescents evidenced mixed results, depending on time spent on social media and active or passive SMU.

The relationship between problematic SMU and well-being in adolescents was also studied by Boer and colleagues [17] in 29 countries participating in the Health Behaviour in School-aged Children survey (HBSC). The results have shown that problematic SMU was related to lower well-being and intense SMU was positively related to specific domains of well-being.

In another systematic review of quantitative and qualitative studies on the relationship between social networks and subjective well-being in adolescents, Webster and colleagues [18] provided evidence of both a positive and negative association between social network use and adolescents’ well-being, with some of the studies providing evidence that online social networks can contribute to well-being, while other studies demonstrated a negative relationship between SMU and well-being. In a scoping review of studies developed before and during COVID-19, the included studies have shown that excessive or non-correct SMU is a risk factor for mental health, including psychological symptoms, alcohol consumption, body image perception, and physical activity [1,19].

Considering adolescents’ perspectives about the association between SMU and mental health and well-being, Popat and Tarrant [20], in a narrative review about adolescents’ perspectives, have identified five main themes, evidencing that self-expression and validation, as well as social engagement and peer support, demonstrated a positive impact on well-being and appearance comparisons, whereas body ideals, pressure to stay connected, and exposure to cyberbullying and harmful content contributed to poor mental health.

Thus, different patterns of SMU seem to involve distinct developmental tradeoffs, in which high active SMU can be expected to negatively predict psychosocial adjustment during early adolescence through adulthood [21]. At the same time, healthy social media use may present benefits for normative development during and after this developmental period. In a longitudinal study carried out with three groups of early adolescents, based on SMU and its impact on psychosocial functioning, Vannucci and McCauley Ohannessian [22] found that the high SMU group presented higher internalizing and externalizing symptoms, higher family conflicts, and lower family and friends support, compared to the High Instagram/Snapchat Use and the Low SMU groups. The authors also found that the High Instagram/Snapchat Use group presented higher delinquent behaviours and school avoidance, compared to the Low SMU group. However, the participants in the High Instagram/Snapchat Use subgroup presented higher close friendship competence and friend support compared to the other two groups.

If it is not surprising that SMU can have a negative impact on well-being and mental health. It is also understandable that SMU can play a beneficial role, allowing adolescents to create online identities, communicate with others, build and maintain social networks, and use social media for expressing themselves and for entertainment. However, the mechanisms underlying individual differences in SMU and in its effects on well-being are still understudied.

Thus, the present study was designed with the main goal of understanding and characterizing, from an ecological perspective, the differences between healthy and less healthy (even dependent) SMU in young people and its influence on adolescents’ well-being. It is expected that the identification of the main contextual and individual factors related to healthy, unhealthy, and dependent SMU can be used as a basis for recommendations to address the impact of the negative risks of SMU and, thus, contribute to the promotion of adolescents’ well-being.

In light of the above, we consider the issue of social media use to be complex, multi-dimensional and fundamental to the health of adolescents. It is important to characterise healthy use, risky use, and dependence on social media in adolescence. Characterising the risk of social media use solely by the time spent using it is reductionist and does not contribute to promoting adolescent health. We therefore aim to understand and characterise the personal, interpersonal, and contextual factors associated with social media use in adolescents, taking into account the level of dependence.

We propose the following research questions:

RQ1—There are statistically significant differences in biopsychosocial health indicators between the different levels of dependence on social networks. Do adolescents with higher levels of dependence on social networks show less positive indicators at the individual, interpersonal and contextual levels?

RQ2—Do the psychological and interpersonal factors differ in the three levels of risk of social network dependency in adolescents?

## 2. Method

### 2.1. Participants

This paper is based on the Health Behaviour in School-aged Children (HBSC) 2022 [7,23,24,25], a survey carried out every 4 years in collaboration with the World Health Organisation (WHO), following an international protocol [26].

The sample includes 7643 students from the 6th, 8th, 10th, and 12th school grades, of which 53.9% were female, with an average age of 15.05 (*SD* = 2.36). The sample is representative of the school grades under study.

### 2.2. Instrument and Procedures

This paper is based on the Health Behaviour in School-aged Children (HBSC) 2022 [7,23,24,25], a survey carried out every 4 years in collaboration with the World Health Organisation (WHO), following an international protocol [26]. In Portugal, this study has been applied since 1998. The instrument used in the Health Behaviour School-Aged Children/WHO study is translated into around 50 languages in a rigorous process regulated by the study’s international team [7,23,24,25]. The Portuguese team follows the required translation and validation procedure. The study has been carried out and coordinated by the same team in Portugal since 1998.

The sample is national, random, representative, and stratified by region of the country (Portugal is made up of 5 regions). The schools and classes to be included in the study are selected at random.

The inclusion criteria are: students in the 6th, 8th, 10th, and 12th grades of Portuguese public education from the selected schools. The students’ parents must sign an informed consent form to authorise their children’s participation. Student participation is voluntary. Non-compliance with the inclusion criteria is an exclusion criterion.

Classes with a total of 10,000 students were selected (2500 students for each year of schooling being analysed), and 7643 valid responses were obtained (the data collection programme only allows the participant to submit the questionnaire if all the questions have been answered, so there are no missing values). A response rate of 76.43 per cent was obtained.

The scale for measuring dependence on social networks is made up of 9 items that assess the difficulties that adolescents experience associated with using social networks, such as “…did you often use social networks to escape from negative feelings?” or “…did you regularly realise that you couldn’t think about anything other than the moment when you could use social networks again?”.

The results of the questions were totaled, and the 3 dependence groups were ob-tained by percentile: No dependence on social networks—Percentile 25%—“minimal dependency”; percentile 25–75%—“moderate dependency”; Percentile 75% “high dependency”.

The kidscreen-10 scale [27] was used to assess wellbeing. It consists of 10 items, for example "did you have enough time for yourself", with a 5-point Likert scale, where 1 is never and 5 is always.

Complementary variables that make up the HBSC/WHO protocol were used, namely Psychophysical Symptoms, Stress Management, online with friends, Screen time during leisure time, Difficulties Relationship with Colleagues, Difficulties Relationship with teachers, Family support, Like school, Discussions with parents and friends, Sleep problems, Violence, Healthy eating, Physical activity, Selfharm, Feel safe at school, Feel safe place where live, Alcohol use and Body perception. More detailed information on the complementary variables can be found at Gaspar et al. [7] and Inchley et al. [23]. The data collected are intended to study adolescents’ behaviours and health habits in their life contexts and their influence on their health/well-being. Up to 21st November 2021, the HBSC/WHO study in Portugal was approved by the Ethics Committee of the Centro Académico de Medicina de Lisboa, Centro Hospital Lisboa Norte (reference no. 281/21) and the General Directorate of Statistics for Education and Science. According to the protocol for applying the Health Behaviour in School-aged Children (HBSC) questionnaire, “cluster sampling” was used for data collection, where the unit of analysis was the class. School clusters voluntarily agreed to participate, and informed consent was obtained from all students’ parents or legal guardians. Responses to the questionnaire were obtained online and anonymously.

### 2.3. Data Analysis

First, a descriptive analysis (mean, standard deviation, and frequency) of the variables included in the study was presented. Next, a comparison was made between the three groups of dependence on the use of social networks (No dependence on social networks; Level of dependence (percentile 25–75%) and Percentile 25% high dependence). To compare the groups, the Chi-square test was used for dichotomous variables and ANOVA Analysis of Variance for comparisons between three or more response hypotheses or scale variables. Finally, linear regression models were carried out with the dependent variable well-being for each of the three social media use dependency groups. To carry out all the analyses, we used the SPSS programme.

## 3. Results

The table below includes information on the variables under study (Table 1).

Comparing the dependency groups in relation to the different variables, statistically significant differences were found in all variables (Well-being, Psychological symptoms, Sleep quality, Physical activity, Healthy eating, Family support, Discussion with family and friends, Relationship with peers and teachers, liking school, Stress management, Violence, SES, and Online relationships), with the exception of the variable Free time spent on screens (Table 2).

A simple linear regression was calculated to understand the prediction of well-being, taking into account the level of dependence on social networks, based on the variables gender, age, FAS, Psychological symptoms, Stress management, Body perception, Self-injury, Physical activity, Online relationships with friends, Free time spent on screens, Healthy eating, Sleep quality, Alcohol consumption, Relationship with peers, Relationship with teachers, Family support, Liking school, Discussion with family and friends, Violence, Perceived safety at school, and Perceived safety in the neighbourhood.

Three different models (Table 3) were analysed for each level of dependence: one for non-dependence on social networks; one for moderate dependence; and one for the 25% percentile of high dependence.

In the first analysis, there was an adjusted model (F = 87.201, *p* ˂ 0.001), with an R2 of 0.62, showing that the total variation in well-being for adolescents without dependence on social networks can be explained by 62% of the independent variable as a whole. In the second adjusted model (F = 204.645, *p* = 0.000), with an R2 of 0.661, showing that the total variation in well-being for adolescents with moderate dependence on social networks can be explained by 66.1% by the independent variable as a whole. The last model, corresponding to high dependence, adjusted (F = 170.871, *p* = 0.000), with an R2 of 0.644, shows that the variation in the well-being of adolescents with high dependence on social networks can be explained by 64.4% by the independent variable as a whole.

The results show that the well-being of adolescents with a higher level of dependence (25% percentile of high dependence) is significantly associated with gender (β = −0.78), age (β = −0.15), psychological symptoms (β = 0.18), stress management (β = 0.70), body perception (β = −0.65), self-injurious behaviour (β = −0.18), physical activity (β = 0.40), online relationships with friends (β = 0.13), free time spent using screens (β = 0.20), alcohol consumption (β = −0.90), relationships with peers (β = −0.15) and teachers (β = −0.16), family support (β = 0.18), and feeling safe at school (β = −0.76) and in the neighbourhood (β = −0.61).

## 4. Discussion

This study pretends to the identity of the main contextual, relational and individual factors related to health, unhealthy and dependent SMU. The main goal was of understanding and characterizing, from an ecological perspective, the differences between healthy and less healthy (even dependent) SMU in young people and its influence on adolescents’ well-being. In the discussion, we will answer and discuss the hypotheses put forward.

We found statistically significant differences between adolescents according to their level of dependence on SMU. Adolescents without SMU dependence are those who show greater well-being, fewer psychological symptoms, more stress management skills, a healthier lifestyle (more physical activity and healthier diet, and more positive family and school relationships), and a higher socioeconomic status. Adolescents with a higher level of SMU dependence are those with more sleep difficulties, more arguments with family and friends, worse relationships with teachers, more involvement in violence, and those spending more time online with friends. There are no statistically significant differences between dependence groups in terms of screen time during free time. The lack of correlation between dependence and screen time may be linked to the fact that the amount of time teenagers spend on screen activities is not the main factor explaining dependence. Nowadays, teenagers use screen activities for a variety of purposes: socialising with friends, playing games, researching, reading, etc. Various activities that used to take many forms are now often carried out with screen gadgets. All these activities are very important for the well-being and healthy development of teenagers, so screen time in itself is not the problem. Psychological and emotional factors, difficulties in relationships with friends and peers, and environmental difficulties (home and neighbourhood) are more relevant factors in understanding social media dependence. However, adolescents with higher dependence spend more free time using screens. Technology and social media use (SMU) is an integral part of adolescents’ lives. We find that it is associated with their well-being, mental health, and with a positive relationship with the peer group and sense of belonging and adaptation [1,3,5].

However, SMU can be considered a risk factor for well-being and mental health, or overuse can be a behaviour that reveals difficulties relating to other contexts in adolescents’ lives. An adolescent with difficulties or a lack of competences in their family, school, and social life may overuse SMU more exclusively as a way to cope with negative feelings [4,20].

What best explains the well-being of all adolescents, regardless of the level of SMU dependence, is age (being younger), having fewer psychological symptoms, having better stress management skills, a positive body perception, more physical activity, being with friends online, having good relationships with teachers, and family support. Being a boy, not self-injuring, not consuming alcohol, and having good relationships with peers help explain the well-being of adolescents with high and moderate dependence. Socioeconomic status, sleep quality, liking school, and having fewer arguments with family and friends are associated with well-being for adolescents with moderate dependence. Well-being for adolescents with high SMU dependence is associated with spending more free time on screen activities and a perception of safety at school and in the neighbourhood.

The results lead us to reflect on the protective factor of ESE, sleep habits, liking school, and positive relationships with family and peers in relation to the intensity of SMU dependence. On the other hand, adolescents with higher dependence are more affected by a lack of safety (school and neighbourhood) and spend more free time using screens, which may remit the use of screens as a way to manage the lack of safety and comfort in activities outside the home.

The relationships found in the proposed multi-dimensional model have been partially identified in various studies, in particular the relationship between the use of social networks and SES. In the study carried out by Arias and colleagues [28] and Männikkö and colleagues [29], they conclude that there is a relationship between higher SES and a greater ability to manage social networks and have a healthier use of them.

Our results also show that the quality of interpersonal relationships is associated with the type of social media use. Interpersonal relationships, especially with parents and friends, appear to be linked to the pattern of social media use. A more positive relationship with parents and friends, more democratic parenting styles, and better communication [30,31] are associated with lower levels of social media dependency.

Sleep habits are fundamental to understanding the healthier or less healthy use of technology. Health behaviours, particularly sleep habits, have been shown to be related to social media use. Hale et al. [32] conclude that there is a relationship between high social media use and sleep difficulties such as delayed bedtime and/or decreased total sleep time.

The contexts in which adolescents live, in particular the school environment and their satisfaction with it, are aspects that should be taken into account when understanding well-being and healthy habits when using social networks. School and connection to school also appear as factors associated with social media dependence. In the study carried out by Khan et al. [33], it was found that teenagers with more screen time showed more stress about school and less satisfaction with and a weaker connection to school. Another study by Peng and colleagues [34] reveals that internet dependence is associated with psychological, social, school, and/or work difficulties and school disconnectedness.

However, our model is innovative in that it understands well-being and dependence on social networks from an ecological perspective.

The main limitations of this paper are that it is a self-report study that may be influenced by social desirability. The SMU measurement instrument is a generalist instrument and is not a clinical instrument for diagnosing dependence, and this is a limitation of this study with its cross-sectional design. Finally, it is a limitation of this study with cross-sectional design, because cross-sectional studies cannot establish a cause-and-effect relationship or analyse behaviour over a period of time. To investigate cause and effect, one needs to carry out a longitudinal study or an experimental study. This paper is a global exploratory study of social networking behaviour. In the HBSC study, we were able to gather a global perspective on adolescent health, which gives us a unique opportunity to analyse this aspect from a very comprehensive perspective. Our aim is not to prove “causality”, but rather an association between variables. This paper provides clues for further in-depth analyses of certain aspects, such as the association with family, psychological factors or even self-image. The scope of the model can be considered a limitation, but also an opportunity to understand the use of social networks by adolescents from a global perspective, prove its complexity, and then delve into it in a more segmented way. The absence of data on sexuality and gender identity as limitations can be considered another limitation because these are also factors in the relationship between SMU and well-being. We have information on nationality, and the gender question has three answer options: boy, girl, or other. The number of respondents for the third option is very small so, as it was not the focus of this paper, we did not include these participants or carry out a comparative analysis. It will be an interesting topic for a future paper.

SMU dependence is a complex phenomenon that is associated with a number of factors; in short, a combination of risk factors, such as insecurity, alcohol consumption, and self-injurious behaviour, and few protective factors, such as liking school and positive relationships with family, friends, and teachers. These results help us understand the similarities and what distinguishes adolescents’ well-being according to the level of dependence. We confirm that adolescent well-being should be viewed from an ecological perspective and that sociodemographic, psychological, social, and contextual factors contribute to this.

## 5. Conclusions

Being online with friends is important for all adolescents, which allows us to suggest that online time is important for the well-being and mental health of adolescents, and it is not the development of online activities that necessarily leads to dependence.

We found that the relationship between SMU and well-being and mental health depends on many factors, from economic factors to psychological factors, to social and environmental factors. More than the amount of time the adolescent spends on social media, we need to check if the use is balanced with other forms of leisure time occupation and the establishment of interpersonal relationships and integration.

The study is relevant and adds to the knowledge about what distinguishes healthy use and dependence on SMU. With the increasing use of technologies for young people’s various activities, such as school, leisure time, and interpersonal relationships, and the prospect that this dynamic will continue and even evolve in the coming years, it is essential to understand that the use of technologies can be healthy if accompanied by protective factors. It is not possible to remove all risk from adolescents’ lives. It will be important to promote protective factors (positive relationships with parents, teachers, friends, perception of safety, alternative leisure activities, etc.) and develop socio-emotional skills for adolescents to manage risks, through self-regulation for healthy choices.

## Figures and Tables

**Table 1:** Descriptive statistics of the variables under study.

Variables	Min	Max	%	*M*	*DP*
Age	10.33	20.17		15.05	2.36
SES	7.00	19.00		13.87	2.19
Less psychophysical Symptoms	11.00	55.00		41.00	10.31
Well-being	12.00	50.00		36.62	7.14
Stress management	4.00	20.00		12.96	3.06
Online with friends	4.00	24.00		15.18	4.41
Screen time during leisure time	3.00	12.00		8.36	1.74
Difficulties in relationships with colleagues	3.00	15.00		6.43	2.55
Difficulties in relationships with teachers	3.00	15.00		6.74	2.54
Family support	4.00	28.00		22.67	6.63
Like school				11.14	2.22
Discussions with parents and friends	13.00	65.00		23.54	9.83
Sleep problems	8.00	24.00		14.48	3.14
Violence	5.00	25.00		5.99	2.15
Healthy eating	4.00	28.00		18.60	4.14
Physical activity	0.00	7.00		3.78	2.04
Self-harm	0	1	21.8		
Feel safe at school	0	1	78.6		
Feel safe at home	0	1	86.2		
Alcohol	0	1	43.3		
Negative body perception	0	1	49.6		
Minimal dependence on social networks	0	2	20.5		
Moderate dependency on social networks	0	2	39.8		
High dependency on social networks	0	2	39.7		

**Table 2:** Comparative analysis of the biopsychosocial and environmental factors according to the level of dependence on social networks.

Variables		%	*M*	*DP*	*X^2^/F*	*Effect Size*
Selfharm	Minimal dependence	8.7			188.82 ***	
	Moderate dependency	21.9				
	High dependency	28.9				
Feel safe at school	Minimal dependence	87.1			83.42 ***	
	Moderate dependency	78.5				
	High dependency	73.8				
Feel safe at home	Minimal dependence	91.6			62.35 ***	
	Moderate dependency	87.1				
	High dependency	82.1				
Alcohol	Minimal dependence	39.3			28.29 ***	
	Moderate dependency	46.8				
	High dependency	41.8				
Negative body perception	Minimal dependence	40.8			60.64 ***	
	Moderate dependency	52.1				
	High dependency	51.6				
Well-being	Minimal dependence		**39.78**	6.39	209.40 ***	0.052
	Moderate dependency		36.11	6.85		
	High dependency		35.50	7.31		
Less psychophysical Symptoms	Minimal dependence		**45.68**	8.63	216.12 ***	0.054
	Moderate dependency		40.04	10.05		
	High dependency		39.54	10.68		
Sleep problems	Minimal dependence		13.32	3.08	125.25 ***	0.041
	Moderate dependency		14.55	2.91		
	High dependency		**15.04**	3.24		
Physical activity	Minimal dependence		**4.11**	2.04	26.09 ***	0.007
	Moderate dependency		3.69	2.00		
	High dependency		3.70	2.06		
Healthy eating	Minimal dependence		**19.36**	4.18	43.32 ***	0.011
	Moderate dependency		18.64	4.02		
	High dependency		18.17	4.19		
Family support	Minimal dependence		**24.61**	5.65	101.080 ***	0.026
	Moderate dependency		22.63	6.48		
	High dependency		21.72	7.02		
Discussions with parents and friends	Minimal dependence		20.94	9.53	161.72 ***	0.052
	Moderate dependency		22.30	8.38		
	High dependency		**26.35**	10.73		
Difficulties in relationships with colleagues	Minimal dependence		5.86	2.45	51.412 ***	0.013
	Moderate dependency		6.55	2.47		
	High dependency		**6.61**	2.62		
Difficulties in relationships with teachers	Minimal dependence		6.11	2.53	67.50 ***	0.017
	Moderate dependency		6.82	2.42		
	High dependency		**7.00**	2.60		
Like school	Minimal dependence		**11.70**	2.30	88.78 ***	0.006
	Moderate dependency		11.25	2.07		
	High dependency		10.70	2.25		
Stress management	Minimal dependence		**14.39**	2.99	240.77 ***	0.059
	Moderate dependency		12.76	3.01		
	High dependency		12.42	3.06		
Violence	Minimal dependence		5.69	1.97	74.63 ***	0.019
	Moderate dependency		5.77	1.67		
	High dependency		**6.36**	2.57		
ESE	Minimal dependence		**14.08**	2.12	10.10 ***	0.003
	Moderate dependency		13.77	2.16		
	High dependency		13.87	2.26		
Online with friends	Minimal dependence		14.69	4.53	13.41 ***	0.003
	Moderate dependency		15.21	4.02		
	High dependency		**15.40**	4.69		
Screen time during leisure time	Minimal dependence		8.31	1.78	1.13 (n.s.)	0.026
	Moderate dependency		8.35	1.63		
	High dependency		8.40	1.82		

Note: *** *p* ˂ 0.001; n.s. (não significative) *p* > 0.05.

**Table 3:** Linear regression of variables for the study of dependence on social networks in relation to the well-being of adolescents.

	Constant	*B*	Error	*Beta*	*t*
Model	Minimal dependence	15.85	2.41	-	6.59 ***
	Moderate dependency	17.90	1.75	-	10.20 ***
	High dependency	18.20	1.83	-	9.95 ***
Gender	Minimal dependence	0.01	0.26	0.00	0.02 (n.s.)
	Moderate dependency	−0.43	0.19	−0.03	−2.28 *
	High dependency	−0.78	0.22	−0.06	−3.64 ***
Age	Minimal dependence	−0.19	0.71	−0.06	−2.71 **
	Moderate dependency	−0.16	0.05	−0.04	−3.06 **
	High dependency	−0.15	0.06	−0.04	−2.58 **
SES	Minimal dependence	0.75	0.06	0.03	1.29 (n.s.)
	Moderate dependency	0.10	0.04	0.03	2.50 *
	High dependency	0.02	0.04	0.01	0.47 (n.s.)
Psychophysical symptoms	Minimal dependence	0.23	0.02	0.32	13.32 ***
	Moderate dependency	0.17	0.01	0.26	14.92 ***
	High dependency	0.18	0.01	0.28	15.46 ***
Stress management	Minimal dependence	0.66	0.05	0.31	13.63 ***
	Moderate dependency	0.71	0.04	0.32	20.14 ***
	High dependency	0.70	0.04	0.29	17.10 ***
Negative body perception	Minimal dependence	−0.74	0.25	−0.06	−3.03 **
	Moderate dependency	−0.78	0.17	−0.06	−4.62 ***
	High dependency	−0.65	0.19	−0.05	−3.36 ***
Self-harm	Minimal dependence	0.02	0.16	0.00	0.10 (n.s.)
	Moderate dependency	−0.23	0.08	−0.04	−2.86 **
	High dependency	−0.18	0.08	−0.03	−2.13 *
Physical activity	Minimal dependence	0.39	0.06	0.13	6.42 ***
	Moderate dependency	0.35	0.05	0.10	7.77 ***
	High dependency	0.40	0.05	0.11	7.89 ***
Online with friends	Minimal dependence	0.10	0.03	0.07	3.41 ***
	Moderate dependency	0.10	0.02	0.06	4.45 ***
	High dependency	0.13	0.02	0.08	5.95 ***
Screen time during leisure time	Minimal dependence	0.10	0.07	0.03	1.44 (n.s.)
	Moderate dependency	0.08	0.05	0.02	1.53 (n.s.)
	High dependency	0.20	0.05	0.05	3.65 ***
Healthy eating	Minimal dependence	0.02	0.03	0.02	0.80 (n.s.)
	Moderate dependency	0.03	0.02	0.02	1.48 (n.s.)
	High dependency	0.03	0.02	0.02	1.32 (n.s.)
Sleep problems	Minimal dependence	−0.01	0.04	−0.01	−0.27 (n.s.)
	Moderate dependency	−0.10	0.03	−0.04	−2.95 **
	High dependency	−0.02	0.03	−0.01	−0.70 (n.s.)
Alcohol use	Minimal dependence	−0.39	0.26	−0.03	−1.49 (n.s.)
	Moderate dependency	−0.43	0.18	−0.03	−2.42 *
	High dependency	−0.90	0.20	−0.06	−4.45 ***
Difficulties in relationships with colleagues	Minimal dependence	−0.05	0.06	−0.02	−0.81 (n.s.)
	Moderate dependency	−0.07	0.04	−0.03	−1.96 *
	High dependency	−0.15	0.04	−0.05	−3.63 ***
Difficulties in relationships with teachers	Minimal dependence	−0.28	0.06	−0.11	−5.04 ***
	Moderate dependency	−0.25	0.04	−0.09	−6.43 ***
	High dependency	−0.16	0.04	−0.06	−3.85 ***
Family support	Minimal dependence	0.15	0.02	0.13	6.11 ***
	Moderate dependency	0.17	0.01	0.17	11.67 ***
	High dependency	0.18	0.02	0.18	11.60 ***
Like school	Minimal dependence	0.13	0.06	0.05	2.30 *
	Moderate dependency	0.16	0.04	0.05	3.78 ***
	High dependency	0.05	0.05	0.02	1.06 (n.s.)
Discussions with parents and friends	Minimal dependence	0.02	0.01	0.02	1.22 (n.s.)
	Moderate dependency	0.03	0.01	0.03	2.55 **
	High dependency	0.01	0.01	0.01	0.75 (n.s.)
Violence	Minimal dependence	−0.00	0.06	0.00	−0.02 (n.s.)
	Moderate dependency	−0.06	0.06	−0.02	−1.15 (n.s.)
	High dependency	−0.04	0.04	−0.01	−0.89 (n.s.)
Feel safe at school	Minimal dependence	−0.42	0.38	−0.02	−1.11 (n.s.)
	Moderate dependency	−0.29	0.21	−0.02	−1.37 (n.s.)
	High dependency	−0.76	0.23	−0.05	−3.25 ***
Feel safe at home	Minimal dependence	−0.53	0.44	−0.02	−1.20 (n.s.)
	Moderate dependency	−0.33	0.25	−0.02	−1.32 (n.s.)
	High dependency	−0.61	0.25	−0.03	−2.41 *

Note: *** *p* ˂ 0.001; ** *p* ˂ 0.01; * *p* ˂ 0.05; n.s. (not significative) *p* > 0.05.

## Data Availability

Data is unavailable due to privacy or ethical restrictions.
